# Associations between stool micro-transcriptome, gut microbiota, and infant growth

**DOI:** 10.1017/S2040174420001324

**Published:** 2021-01-07

**Authors:** Molly C. Carney, Xiang Zhan, Akanksha Rangnekar, Maria Z. Chroneos, Sarah J.C. Craig, Kateryna D. Makova, Ian M. Paul, Steven D. Hicks

**Affiliations:** 1 Department of Pediatrics, Penn State College of Medicine, Hershey, PA, USA; 2 Department of Public Health Sciences, Penn State College of Medicine, Hershey, PA, USA; 3 Department of Biology, Eberly College of Science, Penn State University, University Park, PA, USA; 4 Quadrant Biosciences, Syracuse, NY, USA

**Keywords:** Infant growth, microbiome, miRNAs

## Abstract

Rapid infant growth increases the risk for adult obesity. The gut microbiome is associated with early weight status; however, no study has examined how interactions between microbial and host ribonucleic acid (RNA) expression influence infant growth. We hypothesized that dynamics in infant stool micro-ribonucleic acids (miRNAs) would be associated with both microbial activity and infant growth via putative metabolic targets. Stool was collected twice from 30 full-term infants, at 1 month and again between 6 and 12 months. Stool RNA were measured with high-throughput sequencing and aligned to human and microbial databases. Infant growth was measured by weight-for-length *z*-score at birth and 12 months. Increased RNA transcriptional activity of *Clostridia* (*R* = 0.55; Adj *p* = 3.7E-2) and *Burkholderia* (*R* = −0.820, Adj *p* = 2.62E-3) were associated with infant growth. Of the 25 human RNAs associated with growth, 16 were miRNAs. The miRNAs demonstrated significant target enrichment (Adj *p* < 0.05) for four metabolic pathways. There were four associations between growth-related miRNAs and growth-related phyla. We have shown that longitudinal trends in gut microbiota activity and human miRNA levels are associated with infant growth and the metabolic targets of miRNAs suggest these molecules may regulate the biosynthetic landscape of the gut and influence microbial activity.

## Background

Obesity is a complex, multifactorial disease that results from dysregulated energy intake, expenditure, and storage. Gut microbiota may play an important role in the development and maintenance of obesity^1^. Many studies have reported a decrease in microbiome diversity to be associated with obesity^1–3^. Although Bacteroidetes and Firmicutes phyla have been a major focus of obesity research, recent studies have shown several other families, genera, and species to also be associated with obesity^1,4^. For example, one study found higher concentrations of *Lactobacillus* species and *B. fragilis* in overweight children, compared to their lean peers^4^. Another study found the abundance of *C. histolyticum* and *C. coccoides* to be directly associated with weight reduction in adolescents^5^. The mechanisms underlying these associations may include alterations in host inflammation or direct effects on metabolite production^5,6^. Fungi also play an important role in microbial communities. The gastrointestinal mycobiome is predominantly derived from the Ascomycota and Basidiomycota phyla; however, less abundant phyla have also shown associations with weight changes^7^.

The human microbiome changes rapidly and significantly during the first years after birth, but by age 3 it is largely established and remains relatively stable thereafter^8^. Therefore, infancy and early childhood provide the greatest opportunity for microbiome-related interventions to prevent obesity risk^9,10^. The infant microbiome is associated with growth in the first year of life^11^. This is critical because numerous studies have demonstrated that rapid growth in infancy (defined using either body mass index or weight for length percentiles) increases obesity risk later in life^12–15^.

The infant microbiome is shaped by many factors, including diet, environment, and immune function^8^. The gut micro-transcriptome, defined as the combination of all human small ribonucleic acid (RNA) features present in the gastrointestinal tract, has also been associated with microbiome composition. Many studies focus on the role of micro-ribonucleic acids (miRNAs). These small, non-coding transcripts control gene expression via targeted degradation of messenger RNA (mRNA). miRNAs are involved in modulation of the host immune response and cell metabolism, both of which can impact development of the microbiome^16,17^. Accumulating evidence suggests that miRNAs play an important role in obesity. For example, the miR-30 family has been shown to influence adipocyte development, and miRNA-194 levels have been associated with obesity-related inflammation^18,19^. Other studies suggest that miRNAs may modulate weight through influences on microbial growth and proliferation^20^.

Given the association of weight with both the microbiome and miRNAs, we hypothesized that dynamics in infant miRNA levels and transcriptional activity of stool microbes during the first year after birth would be associated with infant growth. The aim of this study was to identify putative relationships between host miRNA expression and gut microbe activity that might influence infant growth. Although studies have looked at the microbiome and weight status in childhood, to our knowledge, no studies have examined associations between miRNA expression and microbial transcripts in relationship to infant growth. Understanding how interactions between host and microbial factors impact infant growth could enhance our knowledge of the developmental origins of adult obesity.

## Methods

This study was approved by the Institutional Review Board (IRB) at Penn State College of Medicine. Participants were recruited from Penn State Milton S. Hershey Medical Center in Hershey, PA. All participants were provided informed consent before participating in the study.

### Participants

Participating infants were siblings of children who took part in the Intervention Nurses Start Infants Growing on Healthy Trajectories (INSIGHT) study^21^. The INSIGHT study involved a randomized, controlled trial evaluating the efficacy of a responsive parenting intervention for preventing rapid infant weight gain. These second-born infants were recruited for an observational ancillary study exploring the effect of genetics and environment on growth and development. Participants (*n* = 30) included full-term (>38 weeks gestation) singleton infants receiving primary care at a Penn State Health practice site. Exclusion criteria included infant medical conditions that could significantly affect feeding and/or growth (e.g. cleft palate, congenital heart disease), maternal conditions that could affect postpartum care (e.g. narcotic drug use, chemotherapy), intrauterine growth restriction on prenatal ultrasound, birthweight <2500 g, or plans to move out of the Central Pennsylvania area within 1 year of delivery. Families with a predisposition to obesity were not specifically targeted for recruitment. Weight and recumbent length were measured for each infant at birth and again at 12 months by a trained research nurse^21^. Medical and demographic information, including infant sex, race, mode of delivery, duration of breastfeeding, maternal weight, and INSIGHT intervention group (responsive parenting intervention or home safety control) were collected through a combination of maternal survey and chart review (Table 1). The primary medical outcome was infant growth, defined by World Health Organization (WHO) weight-for-length *z*-score from 0 to 12 months.

### Stool collection and processing

Stool samples were collected from 30 infants at age 1 month (*±*1 week) and then again at 6 or 12 months. Stool was self-collected by parents from the diaper using a sterile collection kit tube (Globe Scientific, VWR, Radnor, PA). Samples were immediately frozen in the home freezer and subsequently transported on ice to the research clinic where they were stored at −80°C until processing. RNA was extracted using the Norgen stool total RNA purification kit (Norgen Biotek, Ontario, Canada) per manufacturer instructions. Samples were flash-thawed and 200 mg of stool was weighed for processing. For samples where 200 mg of stool was not available, the maximum amount available was used (median = 197 mg, range = 40 mg–200 mg). After extraction, RNA yield and purity was determined using a Nanodrop Spectrophotometer (Thermo-Fischer Scientific, Waltham, MA, USA). Extracted RNA was stored at −80°C prior to sequencing.

### RNA sequencing

Small RNA sequencing libraries were constructed from 100 ng total RNA using the NEXTflex Small RNA-Seq Kit v3 (BioO Scientific; Austin, TX). The final product was assessed for its size, distribution, and concentration using an Agilent 2100 Bioanalyzer (Agilent Technologies; Santa Clara, CA). The libraries were pooled and diluted to 3 nM using 10 mM Tris-HCl, pH 8.5 and then denatured following the standard Illumina protocol. The denatured libraries were loaded onto a S1 flow cell on an Illumina NovaSeq 6000 (Illumina) and run at a target depth of 10 million small RNA (<40 base-pair) sequencing reads per sample. De-multiplexed sequencing reads were generated using Illumina bcl2fastq (released version 2.20.0.422, Illumina) allowing no mismatches in the index read. After quality filtering, quality trimming, adapter trimming, and read length-based filtering with the FASTX-Toolkit (http://hannonlab.cshl.edu/fastx_toolkit), 4 bases were trimmed from both 5’ and 3’ ends of the sequencing reads by the fastq trimmer function. These pre-processed small RNA sequences were aligned to mature and precursor miRNA databases using miRbase21, and other small RNA transcripts (including mRNA, small nucleolar RNA, ribosomal RNA, and long intergenic non-coding RNA) were aligned to RefSeqv84^22^. The small RNA alignment was carried out using the SHRiMP2 aligner, designed specifically for short read lengths, in commercially available Partek^®^ Flow^®^ software^23^. Collectively, we refer to these human small RNA features as the micro-transcriptome. In order to filter out the entire human component, samples were separately aligned to the whole human genome hg38^24^. The resultant unaligned reads were then aligned to the NCBI microbiome database using k-SLAM software, and taxonomic classification at lower level taxa (genus, species, family, order, class) and phyla levels was determined via the *k*-mer method, as previously described^25^. Of note, this alignment technique measures the transcriptional activity (i.e. RNA production) of gut microbes rather than microbial abundance (as in 16S metagenomics approaches). On average, approximately 24% of pre-processed reads are aligned to the microbiome database. The functional pathways represented by the entirety of microbial transcripts were quantified through alignment with the Kyoto Encyclopedia of Genes and Genomes (KEGG) database. For each category (i.e. precursor miRNAs, mature miRNAs, small RNAs, microbial taxa, and KEGG pathways), raw counts were filtered to include only features with at least 10 raw reads in at least 10% of samples. The remaining features within each category were quantile normalized and scaled (mean-centered and divided by the standard deviation of each variable).

### Statistical analysis

To examine the association between infant growth, gut microbiome activity, and infant micro-transcriptome abundance, the following linear mixed model was used:

$$Y_{ij}=a_{0}+\sum_{k=1}^{7}X_{ijk}a_{k}+Z_{ij}R+b_{i}+e_{ij},$$

where *Y*_*ij*_ is the response variable (weight-for-length *z*-score from 0 to 12 months) and *Z*_*ij*_ is the explanatory variable (stool RNA) of infant *i* at time point *j*(*j* = 1, 2), respectively. The first time point was always 1 month and the second time point was either 6 months or 12 months. The primary analysis goal was to study the association between infants’ weight-for-length *z*-score (Y) and micro-transcriptome or microbiome features (Z). Examples of a feature include a microbial taxon, a microbial KEGG representation, and a micro-transcriptome RNA. When studying the association between the outcome and the micro-transcriptome or microbiome feature, we included seven clinical covariates in the regression model to adjust for potential confounding effects of these clinical covariates (*X*_*ijk*_, *k* = 1, . . . , 7): infant sex, infant race, breastfeeding duration (weeks), delivery mode, antibiotic use at time of collection, age at time of collection, and INSIGHT intervention group. These covariates were selected a priori based on existing literature regarding causal relationships affecting infant growth or microbiome development^8,21,26,27^. To capture the correlation of multiple measurements collected from the same infant at different time points, we used a random effect term *b*_*i*_, which assumes an exchangeable correlation structure and the error terms *e*_*ij*_ are distributed as independent standard normal random variables^28,29^. At each stool collection time point, the regression coefficient *R* measures the association between the response in weight-for-length *z*-score and the explanatory variable (stool RNA), after adjusting for potential confounding effects of clinical covariates. Under this model framework, we tested *H*_0_ : *R* = 0 for the outcome and each explanatory variable and then used Bonferroni correction to adjust the raw *p*-values for multiple testing. Statistical significance was determined by comparing the adjusted *p*-value to the nominal α = 0.05 level. All statistical association analyses were conducted in R-software (https://www.R-project.org/) using the vegan package (for alpha diversity) and the miceadds package (for fitting the statistical model).

### Bioinformatics analysis

Putative mRNA targets for mature miRNAs of interest (i.e. those significantly associated with infant growth) were interrogated with mirPath version 3 in DIANA tools, and enrichment of KEGG pathways by high confidence targets (microT-CDS ≥ 0.99) was reported following application of Fisher’s Exact Test^30^. A complete list of putative targets for both mature and precursor miRNAs of interest was generated with the miRDB target mining tool after excluding miRNAs with > 2000 predicted targets and mRNAs with prediction scores < 60^31^. Functions and putative interactions of these mRNAs were interrogated with String version 11.0^32^. Enrichment for protein-protein interactions (PPI) and gene ontology molecular functions were reported. We used the Taxon Set Enrichment Analysis (TSEA) within MicrobiomeAnalyst to interrogate phenotypes, associations with host genetic variations, and associations with host disease states for growth-associated microbes^33^. The shotgun data profiling tool was used to interrogate KEGG pathways represented by microbial transcripts for enrichment of specific metabolic functions. Multiple testing correction was applied to all bioinformatics results.

## Results

### Participants

Medical and demographic characteristics of the participants are presented in Table 1. The majority were white (93%) and delivered vaginally (73%). Most (60%) of the infants received some breast milk. Duration and exclusivity of breastfeeding varied widely among infants. At birth, the mean weight-for-length *z*-score was −0.49, which increased to a mean *z*-score of 0.66 at 12 months.

### RNA sequencing results

The average nucleic acid concentration was 160 ng/ul with intermediate purity (A260/280 = 1.86 *±* 0.51, A260/230 = 0.938 *±* 0.43). The average read count per sample among the 62 samples was 6.8 × 10^6^ reads (Range: 2.2 × 10^5^ – 1.2 × 10^7^). After filtering out RNA features with sparse representation (raw counts < 10 in ≥90% of samples), there were 499 microbial taxa that met inclusion criteria (≥10 raw reads in ≥10% of samples), representing 140 KEGG pathways and 25 phyla. There were 150 precursor miRNAs, 53 mature miRNAs, and 73 small RNAs (i.e. mRNAs, long intergenic non-coding RNAs, and ribosomal RNAs) that met abundance criteria for analysis. The most abundant human micro-transcriptome feature in infant stool was miR-134–5p (2000 raw reads per sample; 26% of all mature miRNA reads). The most transcriptionally active microbial phylum was Bacteroidetes (5.6 × 10^5^ reads per sample, 47% of all phylum reads).

### Associations between infant growth and the stool microbiome

**Fig. 1.**
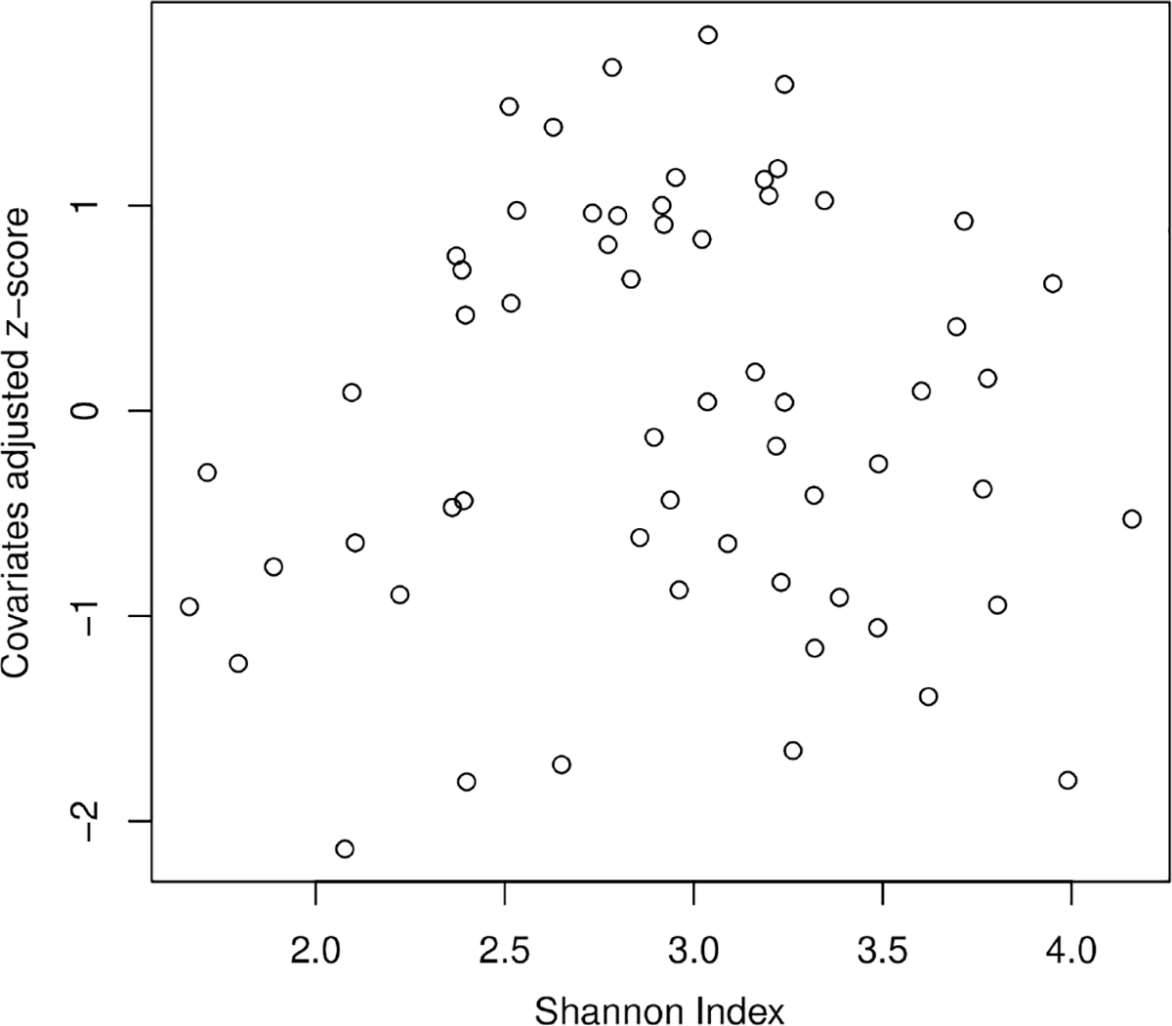
There was no association between alpha diversity of gut microbial transcripts (Shannon Index) and infant growth (weight-for-length z-score from 0 to 12 months).

A linear mixed model was used to examine the association between microbe transcriptional activity and infant growth (weight-for-length *z*-score response), while controlling for infant sex, infant race, breastfeeding duration (weeks), delivery mode, antibiotic use, age, and INSIGHT intervention group. In contrast to prior studies measuring microbial abundance, alpha diversity of microbial transcription products (measured by Shannon Index, Simpson Index, and Inverse-Simpson Index) was not significantly associated with infant growth between 0 and 12 months (Fig. 1). There were 24 out of 499 microbial taxa that demonstrated significant associations (Adj *p* < 0.05) with infant growth (Supplemental Table 1A); transcriptional activity of 14 microbial taxa was positively associated and 10 microbial taxa were negatively associated. Transcriptional activity of Clostridia demonstrated the strongest relationship (*R* = 0.497; Adj *p* = 1.17E-2). At the phylum level, activity of five phyla was significantly associated with infant growth. Two phyla were negatively associated (Cyanobacteria, *R* = −0.273, Adj *p* = 8.1E-3 and Synergistetes, *R* = −0.146, Adj *p* = 1.7E-3) and three phyla were positively associated (Ascomycota, *R* = 0.338, Adj *p* = 6.6E-5; Basidiomycota, *R* = 0.282, Adj *p* = 9.3E-5; and Plantomycetes, *R* = 0.145, Adj *p* = 2.1E-2). Among the 140 KEGG pathways represented by microbial transcripts, three were significantly associated with infant growth (Supplemental Table 1B). The strongest association was observed for K13771 (Rrf2 family transcriptional regulator; *R* = 0.306; Adj *p* = 3.70E-2).

### Associations between infant growth and the stool micro-transcriptome

Among all 276 human RNAs with robust expression in infant stool, 25 were significantly associated (Adj *p* < 0.05) with infant growth (Supplemental Table 1C). In total, 4 features were mature miRNAs, 12 were precursor miRNAs, 3 were long non-coding RNAs, 3 were mRNAs, and 3 were ribosomal RNAs. There were 17 features positively associated and 8 human RNA features negatively associated with infant growth. The human RNA with the strongest growth association was hsa-miR-3613–5p (*R* = 0.456; Adj *p* = 2.31E-7).

### Associations between growth-related microbes and growth-related micro-transcriptome features

There were 1436 significant associations between microbial species and humans RNAs. Among the 24 microbial transcripts that were significantly associated with growth, 5 displayed significant associations with 9 out of the 25 human RNAs associated with growth (a total of 10 significant associations). Analysis of microbial genomes and miRNA seed sequences revealed no sequence homology. There were five significant microbial associations with precursor miRNAs and five with mRNAs (Table 2). *Weissella ceti* activity was the species with the greatest number of significant relationships with growth-related human RNAs (*n* = 5, pre-mir-3689f, Est. = 0.05, Adj *p* = 6.79E-68; SOX2-OT, Est. = 0.86, Adj *p* = 2.51E-5; pre-mir-4505, Est. = 1.03; Adj *p* = 6.41E-122; RPLP0P2 Est. = 1.06, Adj *p* = 2.08E-79; CELF5, Est. = 1.06, Adj *p* = 4.26E-36). Pre-mir-302e was the RNA with the largest number of significant associations with growth-related microbial activity (*n* = 2, *Paenibacillus*, Est. = 0.96, Adj *p* = 4.71E-7, *Scardovia inopinata*, Est. = 0.99, Adj *p* = 7.53E-15). The strongest associations were between *Weissella ceti and* RPLP0P2 (Est. = 1.06, Adj *p* = 2.08E-79) and *Weissella ceti and* CELF5 (Est. = 1.06, Adj *p* = 4.26E-36).

There were 142 significant associations between phyla and human RNAs (Supplemental Table 2a). Of the 5 phyla associated with growth, 4 had significant associations with 1 of the 25 growth-associated human RNAs (a total of four significant associations; Table 3). These RNAs were classified as two precursor miRNAs, one mRNA, and one ribosomal RNA. The strongest association was between Basidomycota activity and pre-mir-7641–1 (*R* = 0.802, Adj *p* = 1.68E-66).

There were a total of 400 significant associations between microbial KEGG pathways and human RNA features (Supplement Table 2b). Two of the three microbial KEGG pathways associated with growth were significantly associated with 7 of the 25 growth-associated human RNAs (a total of eight significant associations; Supplemental Table 3). KEGG pathways were most commonly associated with mature miRNAs (*n* = 3) or precursor miRNAs (*n* = 3).

### Functional analysis

Analysis of the four mature miRNAs associated with infant growth using DIANA miRPATH^30^, identified 46 high-confidence mRNA targets (microT-CDS score ≥ 0.99), with significant enrichment (FDR < 0.05) for four KEGG pathways (Table 4). All four pathways involved biosynthesis/metabolism or degradation/digestion. The most highly enriched pathway was vitamin digestion and absorption (hsa04977; Adj *p* = 2.9E-7), which included a putative interaction between miR-3613–5p and *GIF*. Together, the precursor miRNAs (including both −3p and −5p products) and mature miRNAs displayed seed alignment for 340 mRNAs (based on putative seed matches in miRDB)^31^. Analysis of the protein products of these 340 mRNAs in String software identified 420 edges between 340 nodes, a significant enrichment in protein-protein interactions (PPIs) compared to that expected by chance alone (*p* = 2.50E-4; clustering coefficient 0.368). The PPI network demonstrated enrichment for 19 reactome pathways, particularly those related to NOTCH signaling (Supplemental Table 4).

The microbial features associated with infant growth did not demonstrate enrichment for pathogen/non-pathogen, aerobic/anaerobic, rod/coccus shapes, or gram positive/negative group status (FDR > 0.05; Supplemental Table S5A–C). The set of 41 microbial features displayed nominal associations with genetic variations in SCYL3 (*p* = 3.9E-2), but this did not survive multiple testing correction (Adj *p* > 0.05). The microbial features also demonstrated nominal enrichment for three human medical conditions (atopy, *p* = 1.3E-2, Adj *p* = 1.0; depression, *p* = 2.6E-2, Adj *p* = 1.0; and malodor in children, *p* = 3.8E-2, Adj *p* = 1.0).

## Discussion

This study identifies discrete microbial taxa and human micro-transcripts within stool that are associated with infant growth from birth to 12 months of age. The physiologic targets of these growth-related human RNAs display enrichment for biosynthetic processes, including vitamin digestion and absorption, and glycosphingolipid synthesis (KEGG targets of mature miRNAs). Although this study does not allow us to draw direct conclusions about micro-transcriptome/microbiome interactions, our bioinformatics analysis provides hypothesis-generating results about human and microbial features with strongly associated expression levels, and common physiologic targets.

Since accelerated growth in the first year is associated with increased risk for obesity later in life, significant disruptions in these miRNAs or microbes could serve as obesity risk markers, or even act as potential targets for early life obesity prevention^12–15^. Although a large number of socioeconomic and anthropometric parameters have been associated with accelerated growth in infancy and future risk for obesity, there is currently no lab test that can establish infant risk^26,27^. This study identifies several human RNAs and microbes associated with accelerated growth. These results could inform future studies aimed at developing a stool test to assess obesity risk.

Probiotic use in infants has been studied for the prevention and treatment of a multitude of pathologies, such as necrotizing enterocolitis, infantile colic, and allergy development^34^. Although the association between the microbiome and future risk of obesity is well characterized, no study has examined probiotic use to prevent accelerated growth in infants^8–11,34^. Our findings delineate novel bacteria that could be employed in studies of weight-protective probiotics. We also identify specific miRNAs as novel targets for obesity therapy. For example, deletion of miR-155 has been shown to prevent diet-induced obesity in female mice^35^. It is unclear if similar miRNA manipulations in humans would impact weight, or if oral administration of miRNAs could influence growth through effects on microbial or cellular metabolism.

The majority of associations between microbial taxa and human micro-transcriptome features occurred among precursor miRNAs (5 out of 10 for lower level taxa and 2 out of 4 for phyla). These stem-loop structures do not directly block mRNA translation until they have been processed into mature miRNAs. Precursor miRNAs can be packaged within exosomes, or other vesicles, allowing them to traverse the extracellular space, dock at distant cells, and influence gene expression^36^. In the current analysis, this may explain why: (1) precursor miRNAs were so prevalent within infant stool; and (2) there were few correlations between mature miRNA and mRNA levels in stool.

**Fig. 2.**
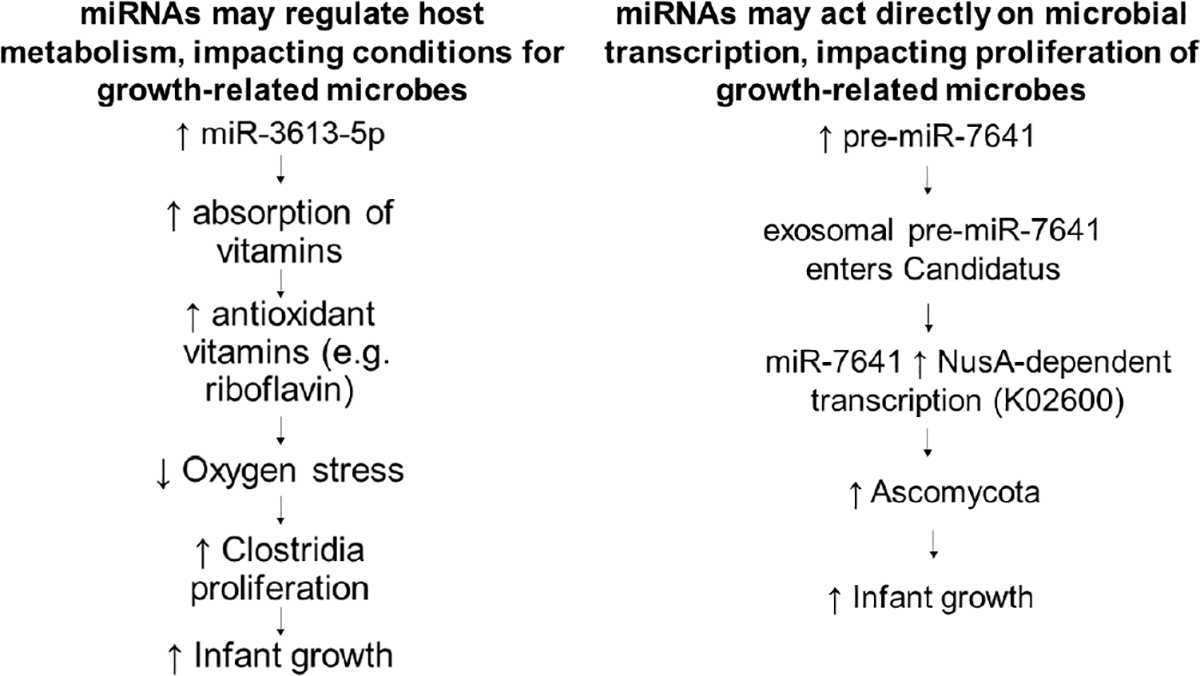
Human microRNAs associated with infant growth may impact microbial activity indirectly (by altering the metabolic landscape), or directly (by entering microbial cells and influencing transcription).

The putative gene targets for growth-related human miRNAs in this study demonstrate robust enrichment for metabolic processes, but show no enrichment for immune-related functions (Table 4). This suggests that miRNAs in the gut predominantly regulate cellular metabolism, rather than immune function. Therefore, observed associations between precursor miRNAs and microbial taxa may result from a commensal shift toward available metabolites, rather than host suppression of particular microbes. This interaction could also explain putative effects of vitamin or probiotic administration on human metabolism vis-à-vis miRNA dynamics. For example, we have shown that the KEGG pathway for vitamin digestion and absorption is highly targeted by miRNA-3613–5p, which is also associated with infant growth. It has been hypothesized that the antioxidant properties of riboflavin may decrease oxygen stress experienced by anaerobic bacteria, resulting in increased proliferation^37^. In fact, riboflavin over-supplementation has been associated with increased levels of *Faecalibacterium prausnitzii* and *Roseburia* species^37^. Both of these species are members of the Clostridia class, which we found to be associated with increased infant growth. Therefore, in the current cohort of infants, we hypothesize that growth-related miRNAs may alter riboflavin metabolism and cause a commensal shift towards Clostridia proliferation and accelerated growth (Fig. 2).

Alternatively, host miRNAs could be affecting the establishment of the microbiome by directly influencing microbial genes. One study showed that human miRNAs produced by the intestinal epithelium and packaged into extracellular vesicles can enter bacteria, co-localize with bacterial nucleic acids, and affect the translation of bacterial growth-related genes^20^. As a result, specific human miRNAs can actually promote the growth of specific microbial species^20,38^. Although the two bacterial species included in this previous study were not found to be associated with infant growth in our study, a similar mechanism could underlie our measured miRNA-microbe associations. For example, the current study identified a direct relationship between Ascomycota and pre-miR-7641 levels. Activity of *Candidatus Kinteoplastibacterium* (a member of the Ascomycota phylum) was directly associated with infant growth. Given that pre-miR-7641 is also associated with the microbial transcription regulator, NusA (K02600), we hypothesize that miR-7641 may impact infant growth through direct effects on Candida transcription and replication (Fig. 2). Notably, previous studies have implicated Candida in type two diabetes^39^, and putative gene targets of miR-7641 (including Pax6) are implicated in maturity onset diabetes of the young^40^.

Some microbes identified in this study have been previously associated with obesity (Ascomycota, Basidiomycota and Clostridia); however, the majority represent novel additions to the scientific literature involving obesity and infant growth^5,7^. Discrepancies with prior literature may arise from our use of an RNA sequencing approach, which reflects microbial transcriptional activity (rather than abundance). This may explain, in part, why we found no relationship between infant growth and the diversity of microbial transcripts, while prior studies have reported a relationship between weight and diversity of microbial abundance (alpha diversity).

There are several limitations of the current study. The sample size (*n* = 62 samples from 30 infants) is small, and prone to type I errors. We have attempted to control for this short-coming through statistical corrections for multiple testing. We view the results as preliminary and hypothesis-generating. The cohort represents a sub-sample from a larger observational study (INSIGHT)^21^. Recognizing that previous psychosocial interventions of INSIGHT could impact growth, we included the parenting intervention group as a covariate in our regression model. The use of longitudinal stool collections in this study is a relative strength. However, the wide age range used for the second stool collection (6–12 months) may have impacted microbial and micro-transcriptome measures. Though all infants had been introduced to solid foods by the time of the second stool collection, the diversity of solid food intake increases drastically between the 6- and 12-month time points^41^. To account for this, age of stool collection was included as a covariate in our analysis. We also controlled for infant sex, infant race, infant age, breastfeeding duration (weeks), delivery mode, and antibiotic exposure, which may impact growth, micro-transcriptomics, or microbial colonization. There are numerous additional factors that could impact infant growth (e.g. timing of solid food introduction, parental body mass index, socioeconomic status, and family dietary habits), and not all of them were available for inclusion in our model. All infants received some breastmilk, thus results may not be generalizable to formula-fed infants. Finally, we note that this cohort is predominantly White. Ethnic influences on diet and genetics may impact both the microbiome and micro-transcriptome, limiting the generalizability of these findings to more ethnically diverse populations.

In conclusion, this study defines both human micro-transcripts and gut microbial transcripts that are associated with growth in infancy. We identify physiologic pathways through which microbes and human hosts may interact to influence metabolism and growth. Though exploratory in nature, this study provides discrete biologic targets for future investigations, allowing researchers to explore mechanisms through which non-coding RNA expression may impact microbial activity and alter biosynthetic processes at the cellular level. Such information might one day be used to generate individualized probiotics or miRNA supplements, and combat rapid growth in infants who are at-risk for obesity later in life.

## Supplementary Material

Supplementary Material

## Figures and Tables

**Table 1.** Medical and demographic characteristics

Characteristic	*n* = 30
Male sex	13 (43%)
White race	28 (93%)
Vaginal Delivery	22 (73%)
Birthweight (kg)	3.5 (±0.4)
WHO weight for length *z*-score at birth	−0.49 (±1.2)
Infant weight at 12 months (kg)	9.6 (±1.0)
WHO weight for length *z*-score at 12 months	0.66 (±0.8)
Maternal pre-pregnancy BMI	26.3 (±4.9)
Duration of Breastfeeding	
No breastfeeding	12 (40%)
3 weeks	3 (10%)
16 weeks	2 (6.7%)
28 weeks	4 (13.3%)
52 weeks	9 (30%)
Responsive parenting study group*	12 (40%)

* Members of the INSIGHT parenting study group received education on responsive parenting and obesity prevention with their first child, while member of the safety “control” group received education on accident prevention.

**Table 2.** Associations between growth-associated microbial lower level taxa and growth-associated micro-transcriptome features

Species	RNA	Estimate	Adj p
Weissella ceti	pre-mir-4505	1.03	6.41E-122
Weissella ceti	RPLP0P2	1.06	2.08E-79
Weissella ceti	pre-mir-3689f	0.05	6.79E-68
Weissella ceti	CELF5	1.06	4.26E-36
Scardovia inopinata JCM 12537	pre-mir-302e	0.99	7.53E-15
Arthrobacter	LSM14B	0.95	2.42E-11
Paenibacillus sp. LPB0068	pre-mir-302e	0.96	4.71E-07
Alistipes	pre-mir-5195	0.66	7.77E-06
Weissella ceti	SOX2-OT	0.86	2.51E-05
Alistipes	CEBPZOS	0.63	6.92E-03

**Table 3.** Associations between growth-associated microbial phyla and growth-associated micro-transcriptome features

Phylum	RNA	Estimate	Adj p
Synergistetes	DBET	0.064	4.03E-70
Basidiomycota	pre-mir-7641-1	0.802	1.68E-66
Ascomycota	pre-mir-7641-1	0.754	2.37E-05
Planctomycetes	RNA28SN3	−0.321	6.00E-04

**Table 4.** KEGG pathways targeted by mature miRNAs associated with infant growth

KEGG pathway	Adj p	#genes	#miRNAs
Vitamin digestion and absorption	2.90E-07	1	1
Glycosphingolipid biosynthesis – ganglio series	3.05E-07	1	1
2-Oxocarboxylic acid metabolism	2.71E-04	1	1
Citrate cycle (TCA cycle)	2.35E-02	1	1
