# Supplementary material for: Associations between stool micro-transcriptome, gut microbiota, and infant growth
Source: J Dev Orig Health Dis. Author manuscript; Available in PMC 2022 Dec 1. (PMC8675179; doi:10.1017/S2040174420001324)
Supplement: Supplementary Material [file NIHMS1760142-supplement-Supplementary_Material.docx]

**Supplementary Table 1A. Microbial taxa associated with infant growth**

| Species | Est | Pvalue | Adj-P |
| --- | --- | --- | --- |
| Bacillus | 0.392 | 5.17E-16 | 2.58E-13 |
| Arthrobacter | -0.302 | 3.14E-12 | 1.57E-09 |
| Thermoanaerobacterales | 0.366 | 8.98E-11 | 4.48E-08 |
| Paenibacillus sp. LPB0068 | -0.265 | 4.87E-08 | 2.43E-05 |
| Pluralibacter gergoviae | -0.305 | 6.78E-08 | 3.39E-05 |
| Candidatus Kinetoplastibacterium oncopeltii TCC290E | 0.239 | 1.59E-07 | 7.91E-05 |
| Weissella ceti | 0.252 | 1.86E-07 | 9.27E-05 |
| Bacillaceae | 0.340 | 5.60E-07 | 2.80E-04 |
| Thermoanaerobacter italicus Ab9 | 0.381 | 2.22E-06 | 1.11E-03 |
| Actinomyces sp. oral taxon 414 | 0.362 | 2.40E-06 | 1.20E-03 |
| Thermoanaerobacterium thermosaccharolyticum DSM 571 | 0.297 | 4.44E-06 | 2.22E-03 |
| Burkholderia | -0.820 | 5.25E-06 | 2.62E-03 |
| Rhizobium | -0.391 | 6.78E-06 | 3.38E-03 |
| Scardovia inopinata JCM 12537 | -0.282 | 2.28E-05 | 1.14E-02 |
| Clostridia | 0.497 | 2.35E-05 | 1.17E-02 |
| Clostridium formicaceticum | 0.318 | 4.02E-05 | 2.00E-02 |
| Turicibacter sp. H121 | 0.232 | 4.30E-05 | 2.15E-02 |
| Alistipes | -0.335 | 4.33E-05 | 2.16E-02 |
| Agrobacterium tumefaciens | -0.433 | 5.01E-05 | 2.50E-02 |
| Clostridioides difficile | 0.294 | 6.83E-05 | 3.41E-02 |
| Caulobacteraceae | -0.467 | 7.13E-05 | 3.56E-02 |
| Selenomonas sp. oral taxon 920 | 0.287 | 7.48E-05 | 3.73E-02 |
| Staphylococcus epidermidis | -0.261 | 7.87E-05 | 3.93E-02 |
| Heliobacterium modesticaldum Ice1 | 0.402 | 9.53E-05 | 4.76E-02 |

**Supplementary Table 1B. Microbial KEGG Pathways associated with infant growth**

| KEGG feature | Name | Pathway | Est | Pvalue | Adj-P |
| --- | --- | --- | --- | --- | --- |
| K18516 | csrC | Biofilm Formation – E.Coli | 0.232 | 1.03E-06 | 1.53E-04 |
| K14227 | tRNA-Ile | Aminoacyl- tRNA biosynthesis | 0.147 | 1.94E-04 | 2.87E-02 |
| K13771 | nsrR | Rrf2 family transcriptional regulator | 0.306 | 2.50E-04 | 3.70E-02 |

**Supplementary Table 1C. Micro-transcriptome features associated with infant growth**

| RNA | Est | Pvalue | Adj-P |
| --- | --- | --- | --- |
| pre-mir-302e | -0.2432 | 3.83E-16 | 1.06E-13 |
| RPLP0P2 (rRNA) | 0.2517 | 2.07E-11 | 5.72E-09 |
| CELF5 | 0.2203 | 4.51E-11 | 1.25E-08 |
| hsa-miR-3613-5p | 0.4560 | 8.36E-10 | 2.31E-07 |
| DBET (lncRNA) | -0.1360 | 1.11E-09 | 3.07E-07 |
| pre-mir-4505 | 0.2390 | 5.52E-09 | 1.52E-06 |
| pre-mir-3689f | -0.1202 | 3.70E-08 | 1.02E-05 |
| CEBPZOS | -0.2676 | 7.89E-08 | 2.18E-05 |
| hsa-miR-4693-3p | -0.1254 | 3.89E-07 | 1.07E-04 |
| LSM14B | -0.2414 | 9.93E-07 | 2.74E-04 |
| pre-mir-4289 | 0.3355 | 1.11E-06 | 3.05E-04 |
| pre-mir-5195 | -0.3013 | 2.58E-06 | 7.11E-04 |
| pre-mir-7641 | 0.2996 | 3.41E-06 | 9.41E-04 |
| RNA45SN2 (rRNA) | 0.2056 | 9.24E-06 | 2.55E-03 |
| pre-mir-663a | 0.2179 | 1.32E-05 | 3.65E-03 |
| hsa-miR-151a-5p | 0.3247 | 1.37E-05 | 3.78E-03 |
| RNA28SN3 (rRNA) | -0.3248 | 1.53E-05 | 4.22E-03 |
| hsa-miR-1273g-3p | 0.2136 | 2.77E-05 | 7.64E-03 |
| MALAT1 (lncRNA) | 0.2435 | 3.99E-05 | 1.10E-02 |
| pre-mir-8086 | 0.2416 | 4.39E-05 | 1.21E-02 |
| SOX2-OT (lncRNA) | 0.2666 | 7.82E-05 | 2.16E-02 |
| pre-mir-1273e | 0.2872 | 1.34E-04 | 3.70E-02 |
| pre-mir-3908 | 0.2785 | 1.54E-04 | 4.26E-02 |
| pre-mir-555 | 0.2200 | 1.74E-04 | 4.81E-02 |
| pre-mir-4668 | 0.2135 | 1.76E-04 | 4.85E-02 |

**Supplementary Table 2A. Associations between microbial phyla and micro-transcriptome features**

| Phylum | RNA | Estimate | Adj-P |
| --- | --- | --- | --- |
| Synergistetes | pre-mir-3648-1 | 0.953 | < 1.00E-100 |
| Synergistetes | pre-mir-3648-2 | 0.953 | < 1.00E-100 |
| Synergistetes | pre-mir-5694 | 0.709 | < 1.00E-100 |
| Synergistetes | pre-mir-598 | 0.749 | < 1.00E-100 |
| Synergistetes | pre-mir-4522 | 0.684 | < 1.00E-100 |
| Basidiomycota | pre-mir-4448 | 0.822 | 4.07E-100 |
| Euryarchaeota | pre-mir-196a-1 | 0.716 | 2.09E-92 |
| Basidiomycota | hsa-miR-486-3p | 0.801 | 2.36E-86 |
| Basidiomycota | hsa-miR-486-3p.1 | 0.801 | 2.36E-86 |
| Basidiomycota | RNA5-8SN4 | 0.755 | 8.71E-81 |
| Basidiomycota | RNA5-8SN4.1 | 0.755 | 8.71E-81 |
| Synergistetes | DBET | 0.064 | 4.03E-70 |
| Basidiomycota | pre-mir-3178 | 0.459 | 6.45E-67 |
| Basidiomycota | pre-mir-7641-1 | 0.802 | 1.68E-66 |
| Spirochaetes | pre-mir-196a-1 | 0.699 | 8.46E-65 |
| Euryarchaeota | pre-mir-200a | 0.511 | 9.75E-60 |
| Basidiomycota | pre-mir-3182 | 0.776 | 4.33E-59 |
| Basidiomycota | hsa-miR-183-5p | 0.441 | 1.21E-44 |
| Spirochaetes | RNR1 | 0.339 | 2.37E-44 |
| Euryarchaeota | RPS12 | 0.408 | 2.99E-32 |
| Spirochaetes | pre-mir-200a | 0.493 | 4.18E-30 |
| Euryarchaeota | RNR1 | 0.341 | 1.48E-29 |
| Basidiomycota | pre-mir-486-2 | 0.699 | 5.62E-28 |
| Ascomycota | pre-mir-4532 | 0.493 | 1.22E-27 |
| Nitrospirae | hsa-miR-3916 | 0.586 | 1.61E-27 |
| Basidiomycota | pre-mir-7-1 | 0.618 | 4.12E-27 |
| Nitrospirae | hsa-miR-30b-5p | 0.512 | 5.96E-27 |
| Nitrospirae | H3F3AP4 | 0.558 | 8.26E-27 |
| Nitrospirae | H3F3AP4.1 | 0.558 | 8.26E-27 |
| Euryarchaeota | pre-mir-181b-2 | 0.408 | 1.83E-24 |
| Basidiomycota | pre-mir-1285-2 | 0.673 | 2.51E-23 |
| Deferribacteres | pre-mir-4419a | 0.445 | 1.32E-21 |
| Synergistetes | pre-mir-589 | 0.288 | 5.64E-21 |
| Ascomycota | pre-mir-1285-2 | 0.695 | 9.03E-21 |
| Spirochaetes | pre-mir-378g | 0.469 | 1.68E-20 |
| Euryarchaeota | pre-mir-378g | 0.468 | 2.52E-19 |
| Basidiomycota | pre-mir-24-1 | 0.327 | 5.23E-18 |
| Basidiomycota | hsa-let-7i-5p | 0.372 | 1.37E-17 |
| Cyanobacteria | pre-mir-584 | 0.903 | 1.38E-17 |
| Euryarchaeota | pre-mir-27a | 0.374 | 3.60E-17 |
| Planctomycetes | pre-mir-30a | 0.319 | 2.42E-16 |
| Tenericutes | pre-mir-650 | 0.813 | 2.51E-16 |
| Ascomycota | pre-mir-619 | 0.567 | 2.84E-16 |
| Spirochaetes | pre-mir-27a | 0.371 | 2.94E-16 |
| Spirochaetes | hsa-miR-186-5p | 0.340 | 1.30E-15 |
| Nitrospirae | pre-mir-6777 | 0.303 | 3.35E-15 |
| Spirochaetes | RPS12 | 0.387 | 6.09E-14 |
| Euryarchaeota | hsa-miR-186-5p | 0.339 | 1.42E-13 |
| Planctomycetes | pre-mir-4778 | 0.429 | 2.14E-13 |
| Planctomycetes | LINC00324 | 0.482 | 1.54E-12 |
| Nitrospirae | pre-mir-1250 | 0.255 | 2.33E-12 |
| Nitrospirae | pre-mir-4804 | 0.290 | 4.83E-12 |
| Basidiomycota | pre-mir-619 | 0.526 | 2.12E-11 |
| Euryarchaeota | pre-mir-7-1 | 0.273 | 4.70E-11 |
| Planctomycetes | pre-mir-203a | 0.483 | 5.61E-11 |
| Basidiomycota | hsa-miR-3135b | 0.473 | 8.35E-11 |
| Spirochaetes | pre-mir-7-1 | 0.264 | 1.62E-10 |
| Basidiomycota | pre-mir-4532 | 0.454 | 3.75E-10 |
| Cyanobacteria | pre-mir-1302-10 | 0.754 | 3.96E-10 |
| Basidiomycota | pre-mir-5095 | 0.538 | 4.41E-10 |
| Euryarchaeota | pre-mir-24-1 | 0.279 | 2.36E-09 |
| Spirochaetes | pre-mir-24-1 | 0.283 | 2.52E-09 |
| Spirochaetes | pre-mir-181b-2 | 0.373 | 3.86E-09 |
| Euryarchaeota | pre-mir-449c | 0.122 | 4.14E-09 |
| Synergistetes | pre-mir-1908 | 0.240 | 8.70E-09 |
| Nitrospirae | hsa-miR-141-3p | 0.458 | 1.77E-08 |
| Cyanobacteria | RPPH1 | 0.739 | 2.94E-08 |
| Synergistetes | pre-mir-3621 | 0.166 | 4.93E-08 |
| Deferribacteres | MTRNR2L8 | 0.821 | 7.22E-08 |
| Synergistetes | hsa-miR-3916 | 0.161 | 9.99E-08 |
| Planctomycetes | hsa-miR-203a-3p | 0.453 | 1.41E-07 |
| Synergistetes | RN7SL1 | 0.138 | 4.99E-07 |
| Tenericutes | pre-mir-222 | -0.316 | 6.27E-07 |
| Basidiomycota | pre-mir-4792 | 0.467 | 7.77E-07 |
| Tenericutes | MALAT1 | 0.591 | 3.61E-06 |
| Nitrospirae | RPL37 | 0.342 | 5.76E-06 |
| Nitrospirae | pre-mir-5694 | 0.211 | 5.91E-06 |
| Basidiomycota | pre-mir-181b-2 | 0.185 | 1.08E-05 |
| Deferribacteres | pre-mir-4485 | 0.139 | 1.29E-05 |
| Nitrospirae | ST3GAL1 | 0.074 | 1.74E-05 |
| Ascomycota | pre-mir-7641-1 | 0.754 | 2.37E-05 |
| Euryarchaeota | pre-mir-5189 | 0.154 | 5.13E-05 |
| Tenericutes | pre-mir-2861 | 0.577 | 6.37E-05 |
| Chrysiogenetes | MSX1 | 0.910 | 1.44E-04 |
| Verrucomicrobia | pre-mir-4804 | 0.626 | 1.92E-04 |
| Euryarchaeota | pre-mir-532 | 0.213 | 2.04E-04 |
| Nitrospirae | pre-mir-6833 | 0.262 | 2.27E-04 |
| Tenericutes | hsa-miR-30a-5p | 0.697 | 3.13E-04 |
| Euryarchaeota | pre-mir-222 | 0.222 | 3.66E-04 |
| Deferribacteres | pre-mir-663a | 0.197 | 4.74E-04 |
| Chrysiogenetes | hsa-miR-4668-5p | 0.770 | 5.09E-04 |
| Planctomycetes | RNA28SN3 | -0.321 | 6.00E-04 |
| Chrysiogenetes | FRY | 0.884 | 6.67E-04 |
| Chloroflexi | hsa-miR-194-5p | -0.321 | 7.01E-04 |
| Chloroflexi | hsa-miR-194-5p.1 | -0.321 | 7.01E-04 |
| Ascomycota | pre-mir-5095 | 0.519 | 7.83E-04 |
| Deferribacteres | PHKB.1 | 0.707 | 1.10E-03 |
| Ascomycota | pre-mir-1273g | 0.269 | 1.26E-03 |
| Chrysiogenetes | EMX2 | 0.547 | 1.45E-03 |
| Basidiomycota | hsa-miR-186-5p | 0.368 | 1.77E-03 |
| Ascomycota | pre-mir-4419a | 0.274 | 1.85E-03 |
| Cyanobacteria | pre-mir-146a | 0.454 | 2.70E-03 |
| Nitrospirae | hsa-miR-4693-3p | 0.048 | 3.98E-03 |
| Fusobacteria | pre-mir-3621 | 0.874 | 4.40E-03 |
| Spirochaetes | pre-mir-222 | 0.219 | 4.55E-03 |
| Euryarchaeota | pre-mir-215 | 0.163 | 5.29E-03 |
| Chloroflexi | pre-mir-215 | -0.228 | 5.46E-03 |
| Chrysiogenetes | SOX2-OT | 0.642 | 6.64E-03 |
| Nitrospirae | hsa-miR-194-5p | 0.202 | 7.14E-03 |
| Nitrospirae | hsa-miR-194-5p.1 | 0.202 | 7.14E-03 |
| Ascomycota | pre-mir-4792 | 0.447 | 8.17E-03 |
| Euryarchaeota | pre-mir-92b | 0.184 | 8.22E-03 |
| Verrucomicrobia | pre-mir-3689f | 0.055 | 8.32E-03 |
| Ignavibacteriae | pre-mir-146a | -0.239 | 8.53E-03 |
| Ascomycota | pre-mir-3178 | 0.425 | 9.03E-03 |
| Ascomycota | hsa-miR-183-5p | 0.407 | 9.55E-03 |
| Cyanobacteria | pre-mir-27a | -0.238 | 9.68E-03 |
| Ignavibacteriae | pre-mir-2861 | 0.601 | 1.02E-02 |
| Ignavibacteriae | pre-mir-30e | -0.198 | 1.25E-02 |
| Ignavibacteriae | pre-mir-181b-2 | -0.205 | 1.31E-02 |
| Deferribacteres | PHKB | 0.584 | 1.43E-02 |
| Ascomycota | hsa-miR-486-3p | 0.723 | 1.54E-02 |
| Ascomycota | hsa-miR-486-3p.1 | 0.723 | 1.54E-02 |
| Ignavibacteriae | hsa-miR-151a-3p | -0.184 | 1.62E-02 |
| Ascomycota | pre-mir-4448 | 0.751 | 1.64E-02 |
| Chloroflexi | hsa-miR-26b-5p | -0.288 | 1.69E-02 |
| Basidiomycota | pre-mir-200a | 0.157 | 1.69E-02 |
| Ignavibacteriae | pre-mir-378g | -0.239 | 1.95E-02 |
| Thermotogae | RNA18SN3 | -0.329 | 2.33E-02 |
| Thermotogae | RNA18SN2 | -0.329 | 2.33E-02 |
| Thermotogae | RNA18SN4 | -0.329 | 2.33E-02 |
| Thermotogae | RNA18SN5 | -0.329 | 2.33E-02 |
| Thermotogae | RNA18SN1 | -0.329 | 2.33E-02 |
| Tenericutes | pre-mir-4709 | 0.396 | 2.42E-02 |
| Basidiomycota | pre-mir-6073 | 0.123 | 2.51E-02 |
| Basidiomycota | pre-mir-4741 | 0.306 | 2.55E-02 |
| Nitrospirae | pre-mir-6073 | -0.094 | 2.88E-02 |
| Nitrospirae | pre-mir-4741 | 0.265 | 2.94E-02 |
| Synergistetes | pre-mir-4756 | 0.168 | 4.18E-02 |
| Ascomycota | pre-mir-3182 | 0.707 | 4.77E-02 |
| Verrucomicrobia | pre-mir-6810 | 0.910 | 4.82E-02 |
| Fusobacteria | hsa-miR-6803-3p | 0.696 | 4.90E-02 |

**Supplementary Table 2B. Associations between microbial KEGG pathways and micro-transcriptome features**

| KEGG | RNA | Estimate | Adj-P |
| --- | --- | --- | --- |
| K02355 | pre-mir-4756 | 0.818 | < 1.00E-100 |
| K02355 | pre-mir-6884 | 0.869 | < 1.00E-100 |
| K02355 | pre-mir-92b | 0.792 | < 1.00E-100 |
| K02355 | pre-mir-1265 | 0.685 | < 1.00E-100 |
| K02355 | pre-mir-449c | 0.830 | < 1.00E-100 |
| K02355 | pre-mir-425 | 0.687 | < 1.00E-100 |
| K02355 | hsa-miR-4756-5p | 0.668 | < 1.00E-100 |
| K02355 | SLC6A20 | 0.457 | < 1.00E-100 |
| K03746 | pre-mir-4756 | 0.821 | < 1.00E-100 |
| K03746 | pre-mir-6884 | 0.870 | < 1.00E-100 |
| K03746 | pre-mir-92b | 0.794 | < 1.00E-100 |
| K03746 | pre-mir-1265 | 0.687 | < 1.00E-100 |
| K03746 | pre-mir-449c | 0.834 | < 1.00E-100 |
| K03746 | pre-mir-425 | 0.700 | < 1.00E-100 |
| K03746 | hsa-miR-4756-5p | 0.681 | < 1.00E-100 |
| K03746 | SLC6A20 | 0.459 | < 1.00E-100 |
| K18516 | MALAT1.1 | 0.887 | < 1.00E-100 |
| K04072 | pre-mir-4307 | 0.670 | < 1.00E-100 |
| K02355 | pre-mir-5189 | 0.703 | < 1.00E-100 |
| K14227 | pre-mir-627 | 0.835 | < 1.00E-100 |
| K02355 | pre-mir-5010 | 0.421 | < 1.00E-100 |
| K02355 | pre-mir-4419b | 0.588 | < 1.00E-100 |
| K03746 | pre-mir-4419b | 0.590 | < 1.00E-100 |
| K04072 | CTC1.1 | 0.532 | < 1.00E-100 |
| K03746 | pre-mir-5189 | 0.703 | < 1.00E-100 |
| K03746 | hsa-miR-489-3p | 0.663 | < 1.00E-100 |
| K02355 | hsa-miR-489-3p | 0.658 | < 1.00E-100 |
| K18516 | hsa-miR-151a-5p | 0.589 | < 1.00E-100 |
| K03746 | pre-mir-5010 | 0.418 | < 1.00E-100 |
| K00134 | CTC1.1 | 0.531 | < 1.00E-100 |
| K03746 | pre-mir-617 | 0.527 | < 1.00E-100 |
| K18516 | MALAT1 | 0.542 | 7.65E-98 |
| K02355 | hsa-miR-335-5p | 0.483 | 2.42E-94 |
| K02355 | EEF1A1 | 0.583 | 8.43E-93 |
| K03664 | pre-mir-638 | 0.509 | 9.11E-87 |
| K03746 | EEF1A1 | 0.591 | 7.21E-86 |
| K02355 | pre-mir-647 | 0.302 | 1.88E-82 |
| K03746 | hsa-miR-335-5p | 0.483 | 3.57E-82 |
| K02946 | RPLP2 | 0.606 | 1.86E-81 |
| K04072 | pre-mir-1275 | 0.445 | 6.91E-79 |
| K01744 | pre-mir-567 | 0.913 | 2.97E-78 |
| K02355 | pre-mir-617 | 0.523 | 6.00E-77 |
| K01744 | pre-mir-566 | 0.908 | 5.09E-76 |
| K04072 | pre-mir-617 | 0.592 | 2.10E-72 |
| K02946 | hsa-miR-186-5p | 0.498 | 1.22E-69 |
| K18516 | RPS27 | 0.534 | 2.34E-69 |
| K00134 | pre-mir-617 | 0.594 | 6.93E-69 |
| K01744 | pre-mir-6774 | 0.682 | 4.64E-67 |
| K03746 | pre-mir-647 | 0.310 | 8.93E-66 |
| K18516 | hsa-miR-27a-3p | 0.617 | 9.54E-66 |
| K18516 | MTRNR2L2 | 0.433 | 7.19E-65 |
| K00616 | hsa-miR-186-5p | 0.498 | 1.23E-64 |
| K01744 | hsa-miR-574-5p | 0.889 | 1.80E-64 |
| K02946 | pre-mir-6846 | 0.501 | 2.51E-63 |
| K00134 | pre-mir-1275 | 0.440 | 3.55E-62 |
| K02355 | pre-mir-6793 | 0.188 | 1.81E-60 |
| K02518 | pre-mir-6810 | 0.863 | 1.13E-59 |
| K01744 | RNA45SN2 | 0.772 | 4.61E-59 |
| K14227 | pre-mir-4668 | 0.787 | 9.31E-59 |
| K02355 | hsa-miR-6870-3p | 0.559 | 8.05E-58 |
| K01834 | AATK | 0.533 | 2.81E-56 |
| K14227 | pre-mir-8054 | 0.616 | 6.09E-56 |
| K18516 | RPL37 | 0.431 | 1.97E-55 |
| K00616 | hsa-miR-203a-3p | 0.398 | 3.92E-52 |
| K03746 | pre-mir-6829 | 0.724 | 6.63E-52 |
| K01744 | pre-mir-1273f | 0.842 | 1.01E-51 |
| K03746 | hsa-miR-6870-3p | 0.558 | 1.96E-51 |
| K03544 | hsa-miR-223-3p | 0.663 | 3.49E-51 |
| K02355 | pre-mir-6829 | 0.714 | 2.96E-49 |
| K18516 | TXNIP | 0.388 | 1.10E-48 |
| K01744 | pre-mir-3908 | 0.795 | 2.77E-48 |
| K03544 | pre-mir-223 | 0.604 | 2.87E-48 |
| K02946 | hsa-miR-203a-3p | 0.393 | 3.88E-48 |
| K14061 | pre-mir-6833 | 0.421 | 1.47E-44 |
| K00600 | pre-mir-196a-1 | 0.614 | 5.55E-43 |
| K00554 | NBPF18P | 0.673 | 1.21E-42 |
| K18516 | RNA28SN5.1 | -0.335 | 3.87E-42 |
| K02838 | pre-mir-1265 | 0.334 | 1.85E-41 |
| K02946 | RNR1 | 0.277 | 5.03E-41 |
| K01744 | pre-mir-1273a | 0.825 | 3.66E-40 |
| K03544 | hsa-let-7f-5p | 0.465 | 1.03E-39 |
| K03544 | hsa-let-7f-5p.1 | 0.465 | 1.03E-39 |
| K04072 | pre-mir-4683 | 0.308 | 4.27E-38 |
| K03746 | pre-mir-4683 | 0.301 | 1.94E-36 |
| K00616 | RPLP2 | 0.595 | 2.72E-36 |
| K00616 | pre-mir-6846 | 0.489 | 3.46E-36 |
| K02946 | hsa-miR-183-5p | 0.170 | 5.27E-36 |
| K04072 | CTC1 | 0.338 | 1.62E-34 |
| K05809 | hsa-miR-6724-5p.3 | 0.791 | 1.81E-34 |
| K00134 | pre-mir-4644 | 0.255 | 2.38E-34 |
| K18516 | RNR2 | 0.357 | 2.75E-33 |
| K00134 | pre-mir-4307 | 0.636 | 2.14E-32 |
| K02886 | pre-mir-4656 | 0.468 | 1.26E-29 |
| K14227 | hsa-let-7f-5p | 0.640 | 6.95E-29 |
| K14227 | hsa-let-7f-5p.1 | 0.640 | 6.95E-29 |
| K14227 | pre-mir-2861 | 0.352 | 1.15E-28 |
| K00134 | RNA28SN5.1 | -0.318 | 5.70E-28 |
| K03674 | pre-mir-6833 | 0.417 | 1.45E-27 |
| K02355 | pre-mir-4683 | 0.292 | 1.48E-27 |
| K03746 | pre-mir-6793 | 0.199 | 1.58E-27 |
| K18516 | RPS29 | 0.397 | 4.14E-27 |
| K03746 | pre-mir-598 | 0.273 | 4.24E-27 |
| K02355 | pre-mir-532 | 0.553 | 1.13E-26 |
| K00616 | pre-mir-222 | 0.283 | 6.19E-26 |
| K02600 | pre-mir-3178 | 0.431 | 7.89E-26 |
| K03746 | pre-mir-532 | 0.564 | 1.36E-25 |
| K18516 | RNA28SN2.1 | -0.337 | 4.66E-25 |
| K03544 | pre-mir-215 | 0.382 | 1.65E-24 |
| K00134 | CTC1 | 0.330 | 2.16E-24 |
| K02355 | hsa-miR-486-5p | 0.260 | 3.89E-24 |
| K02355 | hsa-miR-486-5p.1 | 0.260 | 3.89E-24 |
| K02355 | pre-mir-922 | 0.200 | 1.09E-23 |
| K18516 | SNORD140 | 0.286 | 5.97E-23 |
| K18516 | EEF1A1 | 0.378 | 1.31E-22 |
| K02355 | pre-mir-598 | 0.264 | 6.56E-22 |
| K05809 | pre-mir-4497 | 0.727 | 1.64E-21 |
| K01744 | pre-mir-1268a | 0.667 | 4.61E-21 |
| K03746 | hsa-miR-486-5p | 0.262 | 5.27E-21 |
| K03746 | hsa-miR-486-5p.1 | 0.262 | 5.27E-21 |
| K01744 | HBA2 | 0.527 | 1.71E-20 |
| K01744 | hsa-miR-1273g-3p | 0.722 | 2.31E-20 |
| K03746 | pre-mir-922 | 0.214 | 3.69E-20 |
| K01744 | pre-mir-619 | 0.654 | 3.98E-20 |
| K18516 | pre-mir-4668 | 0.329 | 6.88E-20 |
| K02946 | pre-mir-222 | 0.271 | 8.56E-20 |
| K03664 | hsa-miR-423-5p | 0.277 | 9.28E-20 |
| K01744 | HBA1 | 0.712 | 1.24E-19 |
| K14227 | hsa-miR-186-5p | 0.427 | 1.66E-19 |
| K01744 | SNORD140 | 0.530 | 1.72E-19 |
| K02518 | pre-mir-3689f | 0.050 | 2.17E-19 |
| K18516 | RNA28SN2 | -0.344 | 5.39E-19 |
| K18516 | RNA28SN5 | -0.344 | 5.39E-19 |
| K18516 | RNA28SN1 | -0.344 | 5.39E-19 |
| K00600 | pre-mir-378g | 0.413 | 3.13E-18 |
| K02946 | hsa-let-7c-5p | 0.219 | 3.15E-18 |
| K03746 | pre-mir-3167 | 0.247 | 3.85E-18 |
| K18516 | pre-mir-3908 | 0.262 | 5.03E-18 |
| K14227 | hsa-miR-16-5p | 0.614 | 5.25E-18 |
| K14227 | hsa-miR-16-5p.1 | 0.614 | 5.25E-18 |
| K02355 | hsa-miR-423-5p | 0.213 | 5.66E-18 |
| K03746 | pre-mir-4502 | 0.130 | 6.14E-18 |
| K03664 | pre-mir-6809 | 0.393 | 1.62E-17 |
| K02355 | pre-mir-3167 | 0.246 | 3.13E-17 |
| K00600 | pre-mir-200a | 0.434 | 5.99E-17 |
| K00600 | RPS12 | 0.379 | 1.53E-16 |
| K00415 | FAM198B-AS1 | 4.387 | 1.53E-16 |
| K00554 | pre-mir-558 | 0.344 | 1.85E-16 |
| K00134 | pre-mir-4683 | 0.294 | 2.02E-16 |
| K14227 | pre-mir-4741 | 0.495 | 2.44E-16 |
| K14227 | hsa-miR-6832-3p | 0.313 | 2.55E-16 |
| K00554 | pre-mir-4656 | 0.293 | 5.21E-16 |
| K18516 | H3F3AP4 | 0.255 | 7.12E-16 |
| K18516 | H3F3AP4.1 | 0.255 | 7.12E-16 |
| K03746 | hsa-miR-423-5p | 0.212 | 7.32E-16 |
| K02886 | pre-mir-574 | 0.701 | 9.42E-16 |
| K14223 | pre-mir-3607 | 2.065 | 1.13E-15 |
| K02355 | pre-mir-4502 | 0.127 | 2.34E-15 |
| K02355 | ST3GAL1 | 0.092 | 3.16E-15 |
| K03544 | hsa-miR-16-5p | 0.355 | 5.44E-15 |
| K03544 | hsa-miR-16-5p.1 | 0.355 | 5.44E-15 |
| K03746 | ST3GAL1 | 0.092 | 6.96E-15 |
| K00600 | RNR1 | 0.317 | 7.59E-15 |
| K02886 | pre-mir-922 | 0.597 | 9.40E-15 |
| K01744 | pre-mir-8086 | 0.636 | 1.64E-14 |
| K18516 | pre-mir-4277 | 0.469 | 2.14E-14 |
| K02838 | NBPF18P | 0.637 | 9.32E-14 |
| K18516 | RNA28SN3 | -0.267 | 9.66E-14 |
| K02946 | pre-mir-215 | 0.247 | 1.10E-13 |
| K18516 | FLJ44635 | 0.286 | 1.34E-13 |
| K02600 | RNA5-8SN4 | 0.680 | 5.33E-13 |
| K02600 | RNA5-8SN4.1 | 0.680 | 5.33E-13 |
| K02886 | FLJ44635 | 0.369 | 6.11E-13 |
| K14223 | hsa-miR-6836-3p | 2.005 | 6.38E-13 |
| K18516 | pre-mir-567 | 0.246 | 2.40E-12 |
| K01834 | pre-mir-4804 | 0.514 | 3.52E-12 |
| K04072 | RNA28SN5.1 | -0.298 | 6.76E-12 |
| K03544 | pre-mir-181b-2 | 0.306 | 1.05E-11 |
| K03674 | RPL37 | 0.496 | 1.15E-11 |
| K04072 | pre-mir-4644 | 0.295 | 6.05E-11 |
| K14223 | hsa-miR-486-5p | 1.545 | 7.65E-11 |
| K14223 | hsa-miR-486-5p.1 | 1.545 | 7.65E-11 |
| K05809 | pre-mir-4709 | 0.511 | 1.71E-10 |
| K01744 | RNA28SN2.1 | -0.422 | 2.74E-10 |
| K03746 | pre-mir-655 | 0.278 | 2.80E-10 |
| K04072 | hsa-miR-6832-3p | 0.186 | 3.37E-10 |
| K03664 | hsa-miR-3916 | 0.284 | 4.18E-10 |
| K05594 | ST3GAL1 | 0.135 | 5.08E-10 |
| K18516 | pre-mir-27a | 0.325 | 5.73E-10 |
| K18516 | pre-mir-647 | 0.335 | 6.73E-10 |
| K03664 | pre-mir-92b | 0.193 | 7.08E-10 |
| K02355 | pre-mir-655 | 0.276 | 7.21E-10 |
| K14227 | pre-mir-222 | 0.230 | 7.81E-10 |
| K04072 | hsa-miR-151a-5p | 0.177 | 8.04E-10 |
| K00415 | pre-mir-627 | 2.462 | 1.02E-09 |
| K02600 | pre-mir-4792 | 0.445 | 1.14E-09 |
| K00616 | RNR1 | 0.258 | 1.23E-09 |
| K03664 | pre-mir-30e | 0.216 | 2.09E-09 |
| K04072 | hsa-miR-194-5p | 0.200 | 2.61E-09 |
| K04072 | hsa-miR-194-5p.1 | 0.200 | 2.61E-09 |
| K18527 | pre-mir-601 | 6.922 | 3.40E-09 |
| K00616 | pre-mir-215 | 0.247 | 5.16E-09 |
| K01834 | pre-mir-558 | 0.195 | 5.37E-09 |
| K02946 | pre-mir-1302-10 | 0.297 | 5.91E-09 |
| K01744 | pre-mir-1273g | 0.451 | 7.09E-09 |
| K02886 | pre-mir-4747 | 0.303 | 1.45E-08 |
| K00600 | pre-mir-181b-2 | 0.346 | 1.47E-08 |
| K05809 | pre-mir-647 | 0.302 | 2.08E-08 |
| K14055 | pre-mir-30a | 0.494 | 2.58E-08 |
| K03746 | pre-mir-1257 | 0.169 | 3.02E-08 |
| K02355 | pre-mir-1257 | 0.159 | 3.17E-08 |
| K03544 | hsa-miR-141-3p | 0.517 | 3.25E-08 |
| K02961 | pre-mir-1273g | 0.537 | 3.47E-08 |
| K01744 | pre-mir-1273e | 0.530 | 4.43E-08 |
| K03664 | pre-mir-4502 | 0.120 | 5.68E-08 |
| K03040 | pre-mir-3689f | 0.053 | 8.07E-08 |
| K02600 | pre-mir-4448 | 0.720 | 1.07E-07 |
| K18516 | hsa-miR-151a-3p | 0.252 | 1.23E-07 |
| K02838 | pre-mir-4419b | 0.195 | 1.32E-07 |
| K00616 | hsa-let-7c-5p | 0.206 | 1.70E-07 |
| K00415 | pre-mir-6809 | 4.242 | 1.87E-07 |
| K18516 | LINC00504 | 0.221 | 1.93E-07 |
| K14061 | RPL37 | 0.482 | 2.16E-07 |
| K05809 | pre-mir-320a | 0.521 | 2.58E-07 |
| K05594 | EMX2 | 0.816 | 2.60E-07 |
| K00134 | pre-mir-211 | 0.157 | 2.96E-07 |
| K02518 | pre-mir-601 | 0.229 | 4.24E-07 |
| K05594 | hsa-miR-4668-5p | 1.086 | 4.88E-07 |
| K01834 | pre-mir-922 | 0.605 | 6.23E-07 |
| K14061 | hsa-miR-3960 | 0.565 | 6.31E-07 |
| K05809 | RNA5-8SN4 | 0.217 | 7.36E-07 |
| K05809 | RNA5-8SN4.1 | 0.217 | 7.36E-07 |
| K03746 | hsa-miR-141-3p | 0.393 | 7.77E-07 |
| K00616 | hsa-miR-183-5p | 0.156 | 8.58E-07 |
| K03089 | pre-mir-6718 | 0.698 | 9.99E-07 |
| K14229 | pre-mir-598 | 0.340 | 1.04E-06 |
| K14227 | ST3GAL1 | 0.093 | 1.11E-06 |
| K02355 | hsa-miR-141-3p | 0.393 | 1.37E-06 |
| K00134 | hsa-miR-151a-5p | 0.192 | 1.42E-06 |
| K14227 | hsa-miR-3613-5p | 0.350 | 1.65E-06 |
| K02358 | RNR1 | 0.577 | 2.33E-06 |
| K01744 | pre-mir-4738 | 0.408 | 2.39E-06 |
| K02518 | pre-mir-4804 | 0.570 | 2.41E-06 |
| K03674 | RNA28SN5.1 | -0.432 | 2.74E-06 |
| K03695 | pre-mir-146a | 0.212 | 3.45E-06 |
| K02946 | RPPH1 | 0.267 | 3.86E-06 |
| K01744 | pre-mir-6124 | 0.134 | 5.02E-06 |
| K03664 | RNA5-8SN4 | 0.167 | 5.27E-06 |
| K03664 | RNA5-8SN4.1 | 0.167 | 5.27E-06 |
| K06006 | hsa-miR-16-5p | 0.403 | 5.89E-06 |
| K06006 | hsa-miR-16-5p.1 | 0.403 | 5.89E-06 |
| K14061 | pre-mir-1303 | 0.481 | 5.93E-06 |
| K01979 | pre-mir-4451 | 3.564 | 6.02E-06 |
| K02961 | pre-mir-6786 | 0.827 | 7.87E-06 |
| K01744 | H3F3AP4 | 0.469 | 9.33E-06 |
| K01744 | H3F3AP4.1 | 0.469 | 9.33E-06 |
| K00616 | pre-mir-1302-10 | 0.284 | 1.25E-05 |
| K02886 | pre-mir-4804 | 0.491 | 1.55E-05 |
| K03695 | pre-mir-194-1 | 0.195 | 1.65E-05 |
| K01834 | pre-mir-1272 | 0.414 | 1.85E-05 |
| K00415 | hsa-miR-6870-3p | 2.333 | 1.87E-05 |
| K02838 | hsa-miR-4693-3p | 0.027 | 2.00E-05 |
| K02838 | hsa-miR-6870-3p | 0.283 | 2.18E-05 |
| K02946 | hsa-let-7i-5p | 0.193 | 2.20E-05 |
| K02355 | pre-mir-146a | 0.149 | 2.53E-05 |
| K03040 | pre-mir-6810 | 0.865 | 2.64E-05 |
| K18516 | pre-mir-566 | 0.183 | 2.80E-05 |
| K02886 | AATK | 0.474 | 3.23E-05 |
| K01744 | pre-mir-4785 | 0.235 | 3.53E-05 |
| K18504 | pre-mir-200a | 1.269 | 3.77E-05 |
| K14218 | RN7SL2 | 0.092 | 3.78E-05 |
| K04072 | hsa-miR-6870-3p | 0.178 | 3.83E-05 |
| K02600 | pre-mir-7641-1 | 0.698 | 4.05E-05 |
| K03695 | pre-mir-222 | 0.220 | 4.25E-05 |
| K01744 | RNA28SN3 | -0.356 | 5.09E-05 |
| K14061 | RNA28SN5.1 | -0.424 | 5.27E-05 |
| K02600 | pre-mir-7-1 | 0.553 | 5.46E-05 |
| K05594 | SOX2-OT | 1.012 | 5.65E-05 |
| K00600 | pre-mir-7-1 | 0.219 | 5.97E-05 |
| K18516 | pre-mir-8054 | 0.162 | 6.48E-05 |
| K03746 | pre-mir-146a | 0.137 | 6.72E-05 |
| K03544 | hsa-miR-195-5p | 0.291 | 7.00E-05 |
| K18527 | hsa-miR-4756-5p | 12.759 | 7.16E-05 |
| K01834 | hsa-miR-30d-5p | 0.321 | 7.75E-05 |
| K00616 | pre-mir-146a | 0.160 | 8.33E-05 |
| K04072 | RNA28SN3 | -0.178 | 8.78E-05 |
| K18504 | hsa-miR-223-3p | 1.462 | 8.91E-05 |
| K03674 | hsa-miR-574-5p | 0.356 | 8.91E-05 |
| K02886 | pre-mir-6855 | 0.072 | 9.01E-05 |
| K02961 | hsa-miR-203a-3p | 0.346 | 9.42E-05 |
| K00415 | hsa-miR-489-3p | 2.719 | 1.02E-04 |
| K03674 | hsa-miR-3960 | 0.550 | 1.09E-04 |
| K00415 | pre-mir-6718 | 2.976 | 1.16E-04 |
| K01876 | RNA45SN3 | 0.475 | 1.17E-04 |
| K02838 | pre-mir-3917 | 0.361 | 1.23E-04 |
| K02600 | pre-mir-4532 | 0.439 | 1.48E-04 |
| K06006 | hsa-let-7f-5p | 0.480 | 1.71E-04 |
| K06006 | hsa-let-7f-5p.1 | 0.480 | 1.71E-04 |
| K00616 | RPPH1 | 0.262 | 1.88E-04 |
| K00134 | pre-mir-3661 | -0.157 | 2.01E-04 |
| K02600 | hsa-miR-183-5p | 0.376 | 2.23E-04 |
| K02886 | pre-mir-3125 | 0.332 | 2.33E-04 |
| K01834 | pre-mir-574 | 0.673 | 2.71E-04 |
| K02863 | pre-mir-320a | 0.538 | 2.80E-04 |
| K00616 | pre-mir-27a | 0.143 | 2.90E-04 |
| K05809 | pre-mir-6832 | 0.228 | 3.06E-04 |
| K14061 | pre-mir-3611 | 0.759 | 3.21E-04 |
| K02600 | pre-mir-3182 | 0.672 | 3.68E-04 |
| K03674 | pre-mir-658 | 0.713 | 3.79E-04 |
| K03695 | hsa-miR-223-3p | 0.298 | 4.15E-04 |
| K00600 | pre-mir-27a | 0.308 | 4.71E-04 |
| K14061 | pre-mir-658 | 0.701 | 4.94E-04 |
| K14227 | pre-mir-650 | 0.132 | 5.02E-04 |
| K00134 | pre-mir-5694 | 0.116 | 5.06E-04 |
| K04072 | pre-mir-3661 | -0.148 | 5.45E-04 |
| K01890 | RPS12 | 0.348 | 5.57E-04 |
| K14223 | pre-mir-5195 | 0.968 | 6.25E-04 |
| K03664 | pre-mir-6846 | 0.277 | 6.45E-04 |
| K00134 | RNA28SN3 | -0.179 | 6.51E-04 |
| K14061 | pre-mir-548a-2 | 0.682 | 6.73E-04 |
| K14061 | pre-mir-6130 | 0.218 | 7.37E-04 |
| K03674 | pre-mir-548a-2 | 0.691 | 7.49E-04 |
| K18516 | hsa-miR-4693-3p | 0.023 | 7.94E-04 |
| K02946 | hsa-miR-192-5p | 0.125 | 8.76E-04 |
| K02946 | hsa-miR-215-5p | 0.125 | 8.76E-04 |
| K02961 | hsa-miR-3135b | 0.546 | 9.61E-04 |
| K14223 | hsa-miR-6886-3p | 1.862 | 1.01E-03 |
| K18517 | pre-mir-6832 | 0.321 | 1.07E-03 |
| K18516 | pre-mir-425 | 0.329 | 1.43E-03 |
| K03544 | pre-mir-3648-1 | 0.218 | 1.58E-03 |
| K03544 | pre-mir-3648-2 | 0.218 | 1.58E-03 |
| K02946 | hsa-miR-200c-3p | 0.133 | 2.07E-03 |
| K03674 | RPS12 | 0.399 | 2.09E-03 |
| K00656 | hsa-miR-486-5p | -0.473 | 2.25E-03 |
| K00656 | hsa-miR-486-5p.1 | -0.473 | 2.25E-03 |
| K14061 | hsa-miR-4693-3p | 0.077 | 2.42E-03 |
| K02886 | pre-mir-1272 | 0.409 | 3.01E-03 |
| K14223 | pre-mir-4436b-2 | 1.510 | 3.12E-03 |
| K18515 | pre-mir-24-1 | 0.365 | 3.64E-03 |
| K01462 | pre-mir-4804 | 0.920 | 3.65E-03 |
| K00134 | hsa-miR-6870-3p | 0.171 | 4.19E-03 |
| K14055 | pre-mir-3160-2 | -0.264 | 4.36E-03 |
| K03674 | pre-mir-1303 | 0.534 | 4.40E-03 |
| K01462 | pre-mir-6855 | 0.125 | 4.40E-03 |
| K00600 | pre-mir-24-1 | 0.235 | 4.53E-03 |
| K03695 | pre-mir-200a | 0.299 | 5.00E-03 |
| K02078 | RN7SL2 | 0.111 | 5.14E-03 |
| K03664 | hsa-miR-30b-5p | 0.148 | 5.34E-03 |
| K14229 | pre-mir-4502 | 0.132 | 5.49E-03 |
| K18516 | pre-mir-6829 | 0.214 | 5.81E-03 |
| K02961 | pre-mir-1237 | 0.575 | 5.91E-03 |
| K01744 | pre-mir-3613 | 0.243 | 6.08E-03 |
| K03664 | pre-mir-181b-2 | -0.179 | 6.42E-03 |
| K01834 | pre-mir-663a | 0.405 | 6.51E-03 |
| K14227 | pre-mir-3160-2 | 0.128 | 6.83E-03 |
| K01890 | FLJ44635 | 0.499 | 6.91E-03 |
| K03674 | RPS29 | 0.630 | 7.10E-03 |
| K14230 | pre-mir-5694 | 1.158 | 7.54E-03 |
| K01834 | pre-mir-4656 | 0.425 | 7.69E-03 |
| K04072 | hsa-miR-151a-3p | -0.128 | 9.22E-03 |
| K06078 | pre-mir-30a | 0.438 | 1.01E-02 |
| K05809 | pre-mir-6884 | 0.110 | 1.19E-02 |
| K03674 | MALAT1 | 0.511 | 1.26E-02 |
| K03040 | pre-mir-4286 | 0.266 | 1.33E-02 |
| K00415 | pre-mir-6777 | 2.744 | 1.36E-02 |
| K02838 | pre-mir-5189 | 0.201 | 1.49E-02 |
| K14229 | pre-mir-3167 | 0.376 | 1.78E-02 |
| K03674 | pre-mir-3611 | 0.747 | 1.79E-02 |
| K05809 | hsa-miR-4429 | 0.264 | 1.82E-02 |
| K05809 | pre-mir-4289 | 0.130 | 1.83E-02 |
| K01744 | hsa-miR-151a-5p | 0.378 | 1.93E-02 |
| K00134 | hsa-miR-194-5p | 0.220 | 1.99E-02 |
| K00134 | hsa-miR-194-5p.1 | 0.220 | 1.99E-02 |
| K14061 | pre-mir-1181 | 0.871 | 2.10E-02 |
| K01818 | pre-mir-1268a | 0.184 | 2.27E-02 |
| K00164 | RN7SL2 | 0.106 | 2.31E-02 |
| K02864 | RNA28SN3 | -0.293 | 2.36E-02 |
| K18515 | pre-mir-27a | 0.368 | 2.50E-02 |
| K01876 | pre-mir-4706 | 0.351 | 2.58E-02 |
| K00134 | pre-mir-4502 | 0.071 | 2.60E-02 |
| K14055 | MTRNR2L8 | 0.262 | 3.03E-02 |
| K05809 | pre-mir-4286 | 0.166 | 3.15E-02 |
| K03040 | pre-mir-601 | 0.246 | 3.27E-02 |
| K02600 | pre-mir-5095 | 0.477 | 3.41E-02 |
| K18527 | hsa-miR-486-5p | 8.995 | 3.50E-02 |
| K18527 | hsa-miR-486-5p.1 | 8.995 | 3.50E-02 |
| K14055 | pre-mir-27a | 0.296 | 3.53E-02 |
| K18516 | hsa-miR-423-5p | 0.214 | 3.58E-02 |
| K14229 | EEF1A1 | 0.574 | 3.61E-02 |
| K03746 | pre-mir-6718 | 0.168 | 3.65E-02 |
| K01744 | RNA45SN3 | 0.380 | 3.71E-02 |
| K02961 | pre-let-7c | 0.386 | 3.72E-02 |
| K04043 | MTRNR2L8 | 0.397 | 3.75E-02 |
| K01979 | hsa-miR-1287-5p | 2.520 | 3.79E-02 |
| K02886 | FAM198B-AS1 | 0.221 | 3.80E-02 |
| K03664 | pre-mir-7851 | 0.315 | 3.88E-02 |
| K02078 | SAMD5 | 0.759 | 3.95E-02 |
| K02355 | pre-mir-1272 | 0.066 | 4.48E-02 |
| K05594 | pre-mir-4683 | 0.420 | 4.72E-02 |
| K05594 | MSX1 | 1.139 | 4.74E-02 |
| K02886 | pre-mir-558 | 0.177 | 4.78E-02 |

**Supplementary Table 3. Associations between growth-associated microbial KEGG pathways and growth-associated micro-transcriptome features**

| KEGG | RNA | Estimate | P | Adj- P |
| --- | --- | --- | --- | --- |
| K18516 | MALAT1 | 0.887 | 0.00E+00 | 0.00E+00 |
| K18516 | hsa-miR-151a-5p | 0.589 | 1.53E-134 | 6.26E-130 |
| K14227 | pre-mir-4668 | 0.787 | 2.28E-63 | 9.31E-59 |
| K18516 | pre-mir-4668 | 0.329 | 1.68E-24 | 6.88E-20 |
| K18516 | pre-mir-3908 | 0.262 | 1.23E-22 | 5.03E-18 |
| K18516 | RNA28SN3 | -0.267 | 2.36E-18 | 9.66E-14 |
| K14227 | hsa-miR-3613-5p | 0.350 | 4.04E-11 | 1.65E-06 |
| K18516 | hsa-miR-4693-3p | 0.023 | 1.94E-08 | 7.94E-04 |

**Supplemental Table 4. Reactome pathways enriched by the PPI networks for mRNAs targeted by growth-related miRNAs**

| Pathway Name | Pathway ID | Count in Gene Set | Adj - P |
| --- | --- | --- | --- |
| Noncanonical activation of NOTCH3 | HSA-9017802 | 5 of 8 | 0.0012 |
| NOTCH4 Activation and Transmission of Signal to the Nucleus | HSA-9013700 | 5 of 11 | 0.0019 |
| EPH-ephrin mediated repulsion of cells | HSA-3928665 | 8 of 50 | 0.0019 |
| Regulated proteolysis of p75NTR | HSA-193692 | 5 of 11 | 0.0019 |
| NRIF signals cell death from the nucleus | HSA-205043 | 5 of 16 | 0.0033 |
| NOTCH2 Activation and Transmission of Signal to the Nucleus | HSA-2979096 | 5 of 22 | 0.0099 |
| Cell death signalling via NRAGE, NRIF and NADE | HSA-204998 | 8 of 75 | 0.0099 |
| NOTCH3 Activation and Transmission of Signal to the Nucleus | HSA-9013507 | 5 of 25 | 0.0126 |
| Nuclear signaling by ERBB4 | HSA-1251985 | 5 of 26 | 0.0131 |
| EPH-Ephrin signaling | HSA-2682334 | 8 of 91 | 0.0209 |
| Activated NOTCH1 Transmits Signal to the Nucleus | HSA-2122948 | 5 of 31 | 0.0222 |
| Signaling by NOTCH2 | HSA-1980145 | 5 of 33 | 0.0243 |
| p75 NTR receptor-mediated signalling | HSA-193704 | 8 of 96 | 0.0243 |
| Constitutive Signaling by NOTCH1 HD+PEST Domain Mutants | HSA-2894862 | 6 of 57 | 0.036 |
| Signaling by NOTCH1 HD+PEST Domain Mutants in Cancer | HSA-2894858 | 6 of 57 | 0.036 |
| Constitutive Signaling by NOTCH1 PEST Domain Mutants | HSA-2644606 | 6 of 57 | 0.036 |
| Signaling by NOTCH1 in Cancer | HSA-2644603 | 6 of 57 | 0.036 |
| Signaling by NOTCH1 PEST Domain Mutants in Cancer | HSA-2644602 | 6 of 57 | 0.036 |
| Signaling by ERBB4 | HSA-1236394 | 5 of 42 | 0.0449 |

**Supplemental Table 5A. Microbial feature groups**

|  | Total | Expected | Hits | Raw p | Adj - P |
| --- | --- | --- | --- | --- | --- |
| Probiotics | 14 | 0.149 | 1 | 0.139 | 1 |
| Non Pathogenic | 68 | 0.723 | 1 | 0.523 | 1 |
| Indole producers | 83 | 0.882 | 1 | 0.597 | 1 |
| Facultative | 190 | 2.02 | 1 | 0.883 | 1 |
| Non Motile | 512 | 5.44 | 1 | 0.998 | 1 |
| Non sporulating | 561 | 5.96 | 1 | 0.999 | 1 |
| Rod-shaped | 578 | 6.14 | 1 | 0.999 | 1 |
| Free-living | 885 | 9.4 | 1 | 1 | 1 |
| Mesophile | 987 | 10.5 | 1 | 1 | 1 |
| Gram + | 1010 | 10.7 | 1 | 1 | 1 |

**Supplemental Table 5B. Microbial associations with human disease**

|  | Total | Expected | Hits | Raw p | Adj - P |
| --- | --- | --- | --- | --- | --- |
| Atopy | 1 | 0.0129 | 1 | 0.0129 | 1 |
| Depression (increase) | 2 | 0.0258 | 1 | 0.0256 | 1 |
| Malodor in children (increase) | 3 | 0.0387 | 1 | 0.0382 | 1 |
| Preterm birth (g_increase) | 4 | 0.0516 | 1 | 0.0506 | 1 |
| Atopic dermatitis (Japan) | 5 | 0.0645 | 1 | 0.0629 | 1 |
| Alzheimer's (increase) | 6 | 0.0773 | 1 | 0.075 | 1 |
| Maternal Antenatal infection (increase) | 6 | 0.0773 | 1 | 0.075 | 1 |
| Chronic Obstructive Pulmonary Disease (COPD) | 8 | 0.103 | 1 | 0.0988 | 1 |
| Cystic Fibrosis | 9 | 0.116 | 1 | 0.11 | 1 |
| Hepatitis B (decrease) | 10 | 0.129 | 1 | 0.122 | 1 |
| Colorectal Carcinoma (US and Vietnam, decrease) | 12 | 0.155 | 1 | 0.145 | 1 |
| Resistance to Immune Checkpoint Inhibitors (increase) | 13 | 0.168 | 1 | 0.156 | 1 |
| Type I Diabetes (increased) | 13 | 0.168 | 1 | 0.156 | 1 |
| Uveitis (increase) | 13 | 0.168 | 1 | 0.156 | 1 |
| Type 2 Diabetes (Chinese, increase) | 16 | 0.206 | 1 | 0.188 | 1 |
| Cirrhosis (China, decrease) | 35 | 0.451 | 1 | 0.368 | 1 |
| Liver Cirrhosis (China, decrease) | 52 | 0.67 | 1 | 0.497 | 1 |

**Supplemental Table 5C. Microbial associations with genetic variations in human RNAs**

|  | Total | Expected | Hits | Raw p | Adj - P |
| --- | --- | --- | --- | --- | --- |
| SCYL3 | 5 | 0.342 | 2 | 0.0392 | 1 |
| ACPP | 1 | 0.0683 | 1 | 0.0683 | 1 |
| CYP2A7 | 1 | 0.0683 | 1 | 0.0683 | 1 |
| HERC2 | 1 | 0.0683 | 1 | 0.0683 | 1 |
| MT1A | 1 | 0.0683 | 1 | 0.0683 | 1 |
| NPHS2 | 1 | 0.0683 | 1 | 0.0683 | 1 |
| SERPIND1 | 1 | 0.0683 | 1 | 0.0683 | 1 |
| TAPBP | 1 | 0.0683 | 1 | 0.0683 | 1 |
| TBC1D13 | 1 | 0.0683 | 1 | 0.0683 | 1 |
| TMEM209 | 1 | 0.0683 | 1 | 0.0683 | 1 |
| ZBTB22 | 1 | 0.0683 | 1 | 0.0683 | 1 |
| AAK1 | 2 | 0.137 | 1 | 0.132 | 1 |
| C11orf41 | 2 | 0.137 | 1 | 0.132 | 1 |
| C17orf101 | 2 | 0.137 | 1 | 0.132 | 1 |
| ELP2 | 2 | 0.137 | 1 | 0.132 | 1 |
| IFT43 | 2 | 0.137 | 1 | 0.132 | 1 |
| KLRC4 | 2 | 0.137 | 1 | 0.132 | 1 |
| PSMB7 | 2 | 0.137 | 1 | 0.132 | 1 |
| PYCRL | 2 | 0.137 | 1 | 0.132 | 1 |
| RASGRF1 | 2 | 0.137 | 1 | 0.132 | 1 |
| RPN1 | 2 | 0.137 | 1 | 0.132 | 1 |
| SDK1 | 2 | 0.137 | 1 | 0.132 | 1 |
| SH2D3C | 2 | 0.137 | 1 | 0.132 | 1 |
| SLC25A29 | 2 | 0.137 | 1 | 0.132 | 1 |
| SLC6A12 | 2 | 0.137 | 1 | 0.132 | 1 |
| ADAM22 | 3 | 0.205 | 1 | 0.192 | 1 |
| ALS2CR12 | 3 | 0.205 | 1 | 0.192 | 1 |
| ANKLE1 | 3 | 0.205 | 1 | 0.192 | 1 |
| ATG2A | 3 | 0.205 | 1 | 0.192 | 1 |
| ATG2B | 3 | 0.205 | 1 | 0.192 | 1 |
| AZU1 | 3 | 0.205 | 1 | 0.192 | 1 |
| BAAT | 3 | 0.205 | 1 | 0.192 | 1 |
| BTBD16 | 3 | 0.205 | 1 | 0.192 | 1 |
| C10orf57 | 3 | 0.205 | 1 | 0.192 | 1 |
| C5orf64 | 3 | 0.205 | 1 | 0.192 | 1 |
| FAM48A | 3 | 0.205 | 1 | 0.192 | 1 |
| HIGD1B | 3 | 0.205 | 1 | 0.192 | 1 |
| HTR3C | 3 | 0.205 | 1 | 0.192 | 1 |
| IGFN1 | 3 | 0.205 | 1 | 0.192 | 1 |
| IPO11 | 3 | 0.205 | 1 | 0.192 | 1 |
| LEPR | 3 | 0.205 | 1 | 0.192 | 1 |
| MC5R | 3 | 0.205 | 1 | 0.192 | 1 |
| MERTK | 3 | 0.205 | 1 | 0.192 | 1 |
| OR2D2 | 3 | 0.205 | 1 | 0.192 | 1 |
| PCNXL3 | 3 | 0.205 | 1 | 0.192 | 1 |
| PKD1L1 | 3 | 0.205 | 1 | 0.192 | 1 |
| TUBD1 | 3 | 0.205 | 1 | 0.192 | 1 |
| XYLT1 | 3 | 0.205 | 1 | 0.192 | 1 |
| ZNF469 | 3 | 0.205 | 1 | 0.192 | 1 |
| C8orf73 | 4 | 0.273 | 1 | 0.248 | 1 |
| CROCC | 4 | 0.273 | 1 | 0.248 | 1 |
| EXD3 | 4 | 0.273 | 1 | 0.248 | 1 |
| JPH3 | 4 | 0.273 | 1 | 0.248 | 1 |
| MAT1A | 4 | 0.273 | 1 | 0.248 | 1 |
| OR51S1 | 4 | 0.273 | 1 | 0.248 | 1 |
| PAXIP1 | 4 | 0.273 | 1 | 0.248 | 1 |
| SETX | 4 | 0.273 | 1 | 0.248 | 1 |
| SNX29 | 4 | 0.273 | 1 | 0.248 | 1 |
| TOR2A | 4 | 0.273 | 1 | 0.248 | 1 |
| TXK | 4 | 0.273 | 1 | 0.248 | 1 |
